# Polydioxanone-Based Membranes for Bone Regeneration

**DOI:** 10.3390/polym13111685

**Published:** 2021-05-21

**Authors:** Sybele Saska, Livia Pilatti, Edvaldo Santos de Sousa Silva, Magda Aline Nagasawa, Diana Câmara, Nelson Lizier, Eduardo Finger, Marta Dyszkiewicz Konwińska, Bartosz Kempisty, Samy Tunchel, Alberto Blay, Jamil Awad Shibli

**Affiliations:** 1 M3 Health Ind. Com. de Prod. Med. Odont. e Correlatos S.A., 640 Ain Ata, Jundiaí 13212-213, Brazil; livia.silva@plenum.bio (L.P.); edvaldo.silva@plenum.bio (E.S.d.S.S.); magda.nagasawa@plenum.bio (M.A.N.); samy.tunchel@plenum.bio (S.T.); alberto.blay@plenum.bio (A.B.); 2 Department of Periodontology and Oral Implantology, Dental Research Division, University of Guarulhos, Guarulhos 07023-070, Brazil; 3 Nicell—Pesquisa e Desenvolvimento Ltd.a, 2721 Av. Indianápolis, São Paulo 04063-005, Brazil; diana.camara@nicell.bio; 4 CCB—Centro de Criogenia Brasil, 1861 Av. Indianápolis, São Paulo 04063-003, Brazil; nelson.lizier@nicell.bio; 5 Hospital Israelita Albert Einstein, 627 Av. Albert Einstein, São Paulo 05652-900, Brazil; finger@haoc.com.br; 6 Department of Anatomy, Poznań University of Medical Sciences, 60-781 Poznan, Poland; m.dyszkiewicz@ump.edu.pl; 7 Department of Histology and Embryology, Poznań University of Medical Sciences, 60-781 Poznan, Poland; bkempisty@ump.edu.pl; 8 Department of Veterinary Surgery, Institute of Veterinary Medicine, Nicolaus Copernicus University in Toruń, 87-100 Torun, Poland; 9 Prestage Department of Poultry Science, North Carolina State University, Raleigh, NC 27695-7608, USA

**Keywords:** polydioxanone, membrane, bone regeneration, scaffold

## Abstract

Resorbable synthetic and natural polymer-based membranes have been extensively studied for guided tissue regeneration. Alloplastic biomaterials are often used for tissue regeneration due to their lower immunoreactivity when compared with allogeneic and xenogeneic materials. Plenum^®^ Guide is a synthetic membrane material based on polydioxanone (PDO), whose surface morphology closely mimics the extracellular matrix. In this study, Plenum^®^ Guide was compared with collagen membranes as a barrier material for bone-tissue regeneration in terms of acute and subchronic systemic toxicity. Moreover, characterizations such as morphology, thermal analysis (Tm = 107.35 °C and crystallinity degree = 52.86 ± 2.97 %, final product), swelling (thickness: 0.25 mm ≅ 436% and 0.5 mm ≅ 425% within 24 h), and mechanical tests (E = 30.1 ± 6.25 MPa; σ = 3.92 ± 0.28 MPa; ε = 287.96 ± 34.68%, final product) were performed. The in vivo results revealed that the PDO membranes induced a slightly higher quantity of newly formed bone tissue than the control group (score: treated group = 15, control group = 13) without detectable systemic toxicity (clinical signs and evaluation of the membranes after necropsy did not result in differences between groups, i.e., non-reaction -> tissue-reaction index = 1.3), showing that these synthetic membranes have the essential characteristics for an effective tissue regeneration. Human adipose-derived stem cells (hASCs) were seeded on PDO membranes; results demonstrated efficient cell migration, adhesion, spread, and proliferation, such that there was a slightly better hASC osteogenic differentiation on PDO than on collagen membranes. Hence, Plenum^®^ Guide membranes are a safe and efficient alternative for resorbable membranes for tissue regeneration.

## 1. Introduction

Guided tissue regeneration/guided bone regeneration (GTR/GBR) includes the stimulation of bone formation in bone defects using different types of membranes (resorbable and non-resorbable), associated or not with bone substitutes or grafts [1,2]. This will further provide mechanical support and cellular exclusion at the affected location, where the surrounding healthy tissue will migrate and promote osteogenesis and osteoconduction [1,3]. The use of resorbable materials as membranes significantly reduces the risk of graft-associated long-term complications. Therefore, the ideal biomaterial for barrier membranes should be resorbable, non-immunogenic, and closely mimic the extracellular matrix (ECM) in which morphology will allow cell adhesion, growth, migration, and differentiation [4,5,6,7,8].

Barrier membranes for guided bone regeneration can be based on natural (allograft or xenograft) or synthetic materials. Xenogeneic collagen matrix membranes indicate good tissue regeneration potential since they induce vascularization, cell migration, adhesion, and connective tissue formation [9,10]. However, these membranes are difficult to handle, control, purify, and sterilize, increasing the risk of pathogenic/viral contamination, immunoreactivity [11,12], and severe post-implant inflammation [13]. Several advantages posed by synthetic polymers when compared to natural polymers lie in their reduced immunogenicity and disease transmission potential [14,15].

Polydioxanone (PDO or PDS) is a colorless, crystalline, resorbable synthetic polymer of multiple repeating ether-ester units fully metabolized by the body. It has shown significant promise in tissue-engineering and biomedical applications due to its enhanced mechanical performance, biocompatibility, low inflammatory response, and long-term resorption [16,17,18]. PDO undergoes slow hydrolytic degradation with its metabolites excreted mainly by the kidneys or digested or exhaled as carbon dioxide [16,18]. PDO membranes and scaffolds are histoconductive, stimulating both bone and cartilage regeneration [17,18,19,20,21].

Mesenchymal stem cells (MSCs) are multipotent cells that have the ability to generate several types of cells [22]. Their ease for cultivation and purification makes them ideal for tissue engineering [23]. Human adipose-derived stem cells (hASCs) are a specific subgroup of MSCs currently being tested in many in vitro and in vivo studies regarding regenerative medicine and cell-based therapy for their capacity to differentiate into mesodermal cell lineages, including adipogenic, osteogenic, and chondrogenic cells [24,25,26]. Nevertheless, the inability to predict or control their differentiation once they are implanted poses a significant limitation in tissue-engineering applications [25]. This disadvantage highlights the importance of barrier membranes for cell retention at the defect site as cell growth and differentiation are influenced by the ambient microenvironment, biomaterial, and availability of growth factors, which can all be imbued in the membrane’s material development for optimal directed tissue repair [4,11,26,27]. Previous studies demonstrated that PDO-based materials were an excellent option for scaffold application, mainly to MSC growth such as hASC differentiation into adipogenic, osteogenic, and chondrogenic cells in vitro [4,28,29].

Plenum^®^ Guide is a PDO-based resorbable synthetic membrane used for GTR/GBR, especially for bone and gingival defects (intraosseous defects and periodontal or peri-implant soft tissues). This study investigated systemic effects in vivo and compared hASC growth behavior on Plenum^®^ Guide and collagen membranes. Additionally, characterizations such as morphology, thermal analysis, swelling, and mechanical tests were performed.

## 2. Materials and Methods

### 2.1. Ethical Statement

All animal studies were conducted at MedLab Produtos Diagnósticos Ltd.a, São Paulo, Brazil, using good laboratory practice (GLP) protocols and fully complying with animal welfare regulations. The protocol for adipose-derived stem cell acquisition and purification (ASCs) was approved by the Ethics Committee of the Universidade Federal de São Paulo, São Paulo, Brazil (no. 1119/2018).

### 2.2. Materials

Plenum^®^ Guide (M3 Health Ind. Com. de Prod. Med. Odont. e Correlatos S.A., Jundiaí, Brazil) was used as a PDO-based barrier membrane (experimental group), while Bio-Gide^®^ (Geistlich Pharma AG, Wolhusen, Switzerland), a natural double-sided porcine collagen membrane, was used as the reference biomaterial (control group).

### 2.3. Characterization

The PDO membranes’ structural morphology used for GBR was sputter-coated with a thin conductive carbon layer and then analyzed via scanning electron microscopy (SEM; JSM7500F, JEOL, Tokyo, Japan; 2 kV accelerating voltage). Subsequently, collagen and Plenum^®^ Guide membranes were stained with a 0.5 mg/mL Hoechst solution (Invitrogen-Thermo Fisher Scientific, Waltham, MA, USA) for 1 min and analyzed via fluorescence microscopy. Image analysis was performed using a ZOE Fluorescent Cell Imager (Bio-Rad, Hercules, CA, USA).

Differential scanning calorimetry (DSC) was performed as a mean to determine the membrane crystallinity degree. All samples were tested in DSC-60 from Shimadzu under a nitrogen atmosphere (flow rate: 50 mL/min) at a heating rate of 10 °C/min. In the first cycle, membranes were submitted to a uniform heating ramp from −10 to 120 °C. After an isotherm of 5 min at 120 °C, samples were heated from −20 °C to 350 °C. Thermal transitions and enthalpy of fusion were obtained from DSC curves in the second heating and temperatures were taken at the peak maximum. The crystallization temperature (Tc), the melting temperatures (Tm), and degree of crystallinity (Xc) of the samples were then determined. The crystallinity was calculated according to Equation (1), where ΔH_m_ is melting enthalpy of sample and ΔH_m_^0^ is melting enthalpy of 100 % crystalline PDO (ΔH_m_^0^ = 141.18 J/g) [30].

%Xc = (∆H_m_/∆H_m_^0^) × 100% (1)

Based on ASTM D882: 2012—“Standard Test Method for Tensile Properties of Thin Plastic Sheeting” [31]—tensile properties were obtained using Instron model 5569 universal testing machines with load cell of 500 N and test speed of 50 mm/min.

Water absorption capacity was evaluated for two different thicknesses of PDO membranes: 0.25 mm and 0.5 mm. The methodology used was based on ASTM D570—“Test Method for Water Absorption of Plastics” [32]. PDO membranes, sterilized or not by ethylene oxide (EtO), were separately immersed in distilled water at room temperature. The samples were removed from distilled water at immersion times of 1 min, 5 min, 10 min, 2 h, and 24 h. After removal, samples were laid on a filter paper so that the excess superficial water could be removed and then weighed. The content of distilled water into the swollen membranes was calculated using Equation (2), where W_d_ represents the weight of the dry membrane and W_s_, the weight of the swollen membrane. All experiments were performed five times per sample.

Water uptake (%) = [W_s_ − W_d_)]/W_s_] × 100 (2)

### 2.4. Biological Evaluation In Vitro

#### 2.4.1. ASC Isolation and Expansion

A fragment of the hypodermal adipose tissue was collected using a dermal punch (0.5 mm) on a serum-free basal culture medium (DMEM/Ham’s F-12, Gibco™, Thermo Fisher Scientific, Waltham, MA, USA). It was washed twice with a phosphate-buffered saline (PBS) solution (at 400 g for 5 min), transferred to a T-25 flask, and incubated with a basal culture medium (10% bovine serum containing 5 µg/mL gentamycin) at 37 °C in a 5% CO_2_ humidified atmosphere. After 3–5 days, the medium was exchanged with a fresh complete culture medium. The cells were then resuspended in 1 mL of complete medium and stained with 0.4% Trypan Blue solution (Sigma Aldrich) and counted using the TC20 Automated Cell Counter (Bio-Rad Laboratories). At 70–80% confluency, the culture was expanded to T-75 flasks (cell density ≥ 5 × 10^3^ cells/cm^2^) [33]. To prevent contamination, microbiological tests were performed for aerobic and anaerobic bacteria and fungi using the BD Bactec Peds PlusTM medium (BD, Franklin Lakes, NJ, USA).

#### 2.4.2. Cell-Integration Capacity

The PDO- and the collagen-based membranes were cut into four pieces of equal size (0.5 cm^2^) for the hASC integration analysis. Each piece was hydrated in the culture medium, seeded with 1 × 10^6^ cells/membrane, and incubated in 35 mm culture plates with the complete culture medium for 11 days (37 °C and 5% CO_2_), with medium exchange after every 3–4 days. At the end of this period, these membranes with hASCs were initially fixed in 2.5% glutaraldehyde and then metallized with gold for SEM analysis.

#### 2.4.3. Cell Migration

Cell migration on different membranes was evaluated. The hASCs were seeded at a density of 4 × 10^5^ cells/membrane and incubated in 35 mm culture plates with the complete culture medium for 21 days (37 °C and 5% CO_2_), with medium exchange every 3–4 days [34]. Subsequently, these membranes were transferred to fresh 35 mm culture plates and treated for cell adhesion. After 24 h, the hASCs that migrated from the membranes and adhered to the plates were quantified and visualized [35].

#### 2.4.4. Cell Viability

To determine cell viability, the hASCs were seeded on PDO and collagen membranes (10^6^ cells/membrane) in 35 mm plates with the complete culture medium and then incubated for 24 h (37 °C and 5% CO_2_). The control sample did not contain any membrane. After 24 h, trypsinized cultures were collected and, after centrifugation (400× *g* and 5 min), the cells were resuspended in 0.5 mL of the complete culture medium and stained with 0.4% Trypan Blue to count viable and non-viable cells that detached from the membrane using a TC20 Automated Cell Counter (Bio-Rad Laboratories, Hercules, CA, USA). The viability was determined in terms of the percentage of viable cells with respect to the total number of cells. The toxicity was determined as follows:

Toxicity = (Control Viability − Membrane viability)/Control Viability(3)

#### 2.4.5. Fluorescence Staining

The distribution and cellular behavior of the hASCs grown on the PDO membranes were evaluated via fluorescence microscopy. According to the manufacturer’s instructions, before the cell culture was performed on the membrane, cells were labeled with a vital fluorescent dye, PKH26 (general PKH26-GL cell connection kit, Sigma Aldrich, St. Louis, MI, USA). Membranes seeded with 2 × 10^5^ cells/mL were stained with the fluorescent marker and cultured in 35 mm culture plates for a period of 4 and 24 h. Membrane fibers were stained blue with a 0.5 mg/mL Hoechst solution (Invitrogen-Thermo Fisher Scientific, Waltham, MA, USA) for 1 min. Later, cells seeded on the PDO membranes were fixed with 4% paraformaldehyde. Cells were permeabilized with 0.01% Triton X-100 diluted in PBS for 5 min for cytoskeleton evaluation. In order to analyze actin filaments, cells were stained with phalloidin-fluorescein isothiocyanate (FITC) for 45 min, while cell nuclei were stained with Hoechst solution. An image analysis was carried out on a ZOE Fluorescent Cell Imager (Bio-Rad, Hercules, CA, USA).

#### 2.4.6. The hASC Differentiation on Membranes

The hASCs were seeded on PDO and collagen membranes (10^6^ cells/membrane) in 35 mm plates with a cell-differentiation inductor medium, i.e., the StemPro^®^ Osteogenesis Differentiation Kit (Gibco™, Thermo Fisher Scientific, Waltham, MA, USA). After 11 days, with medium exchange after every 3–4 days, the membranes were fixed in 2.5% glutaraldehyde and metallized with gold to evaluate their morphology.

### 2.5. Biological Evaluation In Vivo

#### 2.5.1. Sample Preparation for Systemic Toxicity Assays

To prepare samples for systemic toxicity assays, a membrane sample (6 cm²) was extracted at 37 °C for 72 h in a shaker incubator at 100 rpm in 10 mL of a 0.9% sodium chloride solution.

#### 2.5.2. Systemic Toxicity Assay

The systemic toxicity was analyzed according to the ISO 10993-11 protocol [36]. A single dose of 50 mL/kg PDO membrane extract was injected intravenously in the caudal veins of female mice (Mus musculus—Swiss albino mice) in the experimental group (*n* = 5) while 0.9% NaCl was applied in the control group (*n* = 5). The animals were observed 1, 24, 48, and 72 h after application to evaluate the presence of toxic signs (changes in the skin, eyes, respiratory system, cardiovascular and gastrointestinal systems, motor activity, salivation, convulsions, hair erection, weight loss, and/or death).

#### 2.5.3. Subchronic Systemic-Toxicity Assay

This assay was carried out according to the ISO 10993-11 [36] and ISO 10993-6 [37] protocols. The PDO membrane (Plenum^®^ Guide) was implanted in the tibia of rabbits (Oryctolagus cuniculus—New Zealand). Each animal presented an implantation site on the right tibia, with 12 rabbits in the experimental group (6 males and 6 females), and 11 rabbits in the control group (five males and six females). The control group with collagen membranes (Bio-Gide^®^) was used as the reference.

Bone defects were generated with a surgical drill (3 mm in diameter; MPolachini, Brazil) with copious saline irrigation, drilling into the full cortical thickness and exposing the bone marrow. During this operation, the animals were anesthetized (using a combination of xylazine and ketamine) and intramuscularly treated with an analgesic (tramadol). Shortly before the procedure, the animals received local anesthesia in the right tibia (articaine, 4%, 1:100,000). With an appropriate motor (Driller Baby 20:1) and specific surgical cutters, a circular fragment measuring 3 mm in diameter was removed from the cortical bone layer. In the experimental group, the defects were filled with a blood clot and covered with a PDO membrane. In the control group, defects were filled with a blood clot and covered with a collagen membrane. Both types of membranes were cut to a final size of 1.5 cm^2^ before implantation. The flaps were sutured with 4–0 mononylon sutures (Ethicon, Johnson & Johnson, Brazil) and the surgical wounds were cleaned with 2% chlorhexidine, covered with iodopovidone, and protected with a gauze and adhesive dressing. Following the procedure, the animals were treated with anti-inflammatory (Meloxicam) and antibiotic (Pentabiotic) agents for three days so that the pain and the risk of wound infection could be minimized. The treated animals were evaluated for a period of 12 weeks.

#### 2.5.4. Clinical Evaluation

Animals were evaluated for clinical signs of toxicity over a period of three months. After this period, the animals were anesthetized (xylazine and ketamine) to collect blood and later euthanized by anesthetic deepening. They were then submitted to necropsy and organ collection, i.e., liver, spleen, left kidney, left adrenal, testicles/ovaries, proximal lymph node (popliteal), and distal (mesenteric) and implantation sites. The liver, kidney, and spleen were weighed for absolute and relative weights.

#### 2.5.5. Histological Evaluation

Tissues and organs fixed in 10% formaldehyde were decalcified in ethylenediaminetetraacetic acid (EDTA; 0.5 M and pH 8.0) for 10 days and embedded in paraffin blocks. The blocks were cut to 4 μm-thick sections and colored with hematoxylin and eosin (H&E). For semiquantitative evaluation of the bone tissue, five fields (400×) were evaluated in the control and experimental animals. The slides were evaluated according to the parameters and criteria established in Table 1.

Thereafter, the obtained data were transferred to a semiquantitative classification system. The results of the bone neoformation, proliferation, and tissue responses were added at each site to yield the “Total”. The “Total” was calculated using Equation (4):

Individual Total = 2(bone neoformation + proliferation)_tissue_ + tissue response (4)

Finally, the totals across the groups were added (“Group Total”) and divided by the total number of evaluated sites (group mean). The mean or tissue-reaction index was quantified and defined as the difference between the mean values of both groups. The tissue-reaction index was classified as follows: non-reactive: 0–2.9; slightly reactive: 3.0–8.9; moderately reactive: 9.0–15.0; severely reactive: >15.

#### 2.5.6. Blood Evaluation

Blood samples collected before euthanasia were used for blood-count analysis (automated and manual reading), blood-clotting tests (thromboplastin (TPL) and ellagic acid (APTT), and blood-biochemistry analysis.

### 2.6. Statistical Analysis

Using the Lilliefors test, subchronic systemic toxicity data were analyzed for normality. For the parametric data, a Student’s *t*-test was conducted to compare the experimental and control groups, while the Mann-Whitney test (Wilcoxon Rank-Sum test) was utilized for nonparametric data. All seeding experiments were performed twice in at least two independent experiments. Cell-viability mechanical traction properties and crystallinity degree data were analyzed using the Student’s *t*-test. Statistical analysis was performed using the BioEstat 5.3 software (Univ. Estadual do Pará, Pará, Brazil) (*p* < 0.05).

## 3. Results

### 3.1. Membrane Characterization

**Figure 1:**
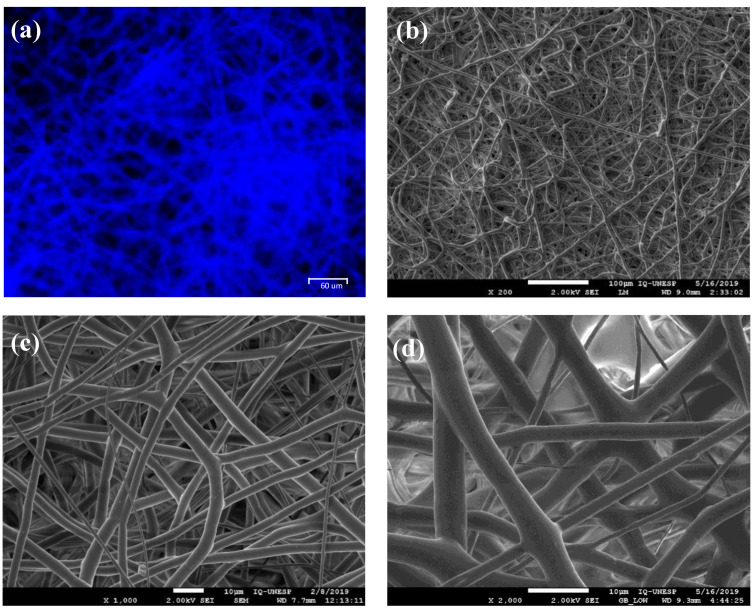
Morphological analysis of the PDO membranes. (**a**) Fluorescence microscopy using Hoechst dye. SEM images at (**b**) 200×, (**c**) 1000×, and (**d**) 2000× magnification.

The superficial topography of the PDO membranes is illustrated in Figure 1. The corresponding SEM and fluorescence images suggest a morphological structure with randomly oriented fibers at the (sub)micron level, similar to the ECM.

PDO is a semicrystalline polymer. The membrane melting temperature (T_m_), the melting enthalpy (ΔH_m_), and the crystallinity degree were determined via differential scanning calorimetry (DSC) (Table 2). The crystallinity degree of the membranes did not show significant statistical differences before and after the sterilization process (Student’s *t*-test, *p* > 0.05). The sterilization by ethylene oxide gas was chosen since the heat treatment or gamma irradiation processes might damage the mechanical properties of PDO because of thermal degradation or thermal oxygen degradation. Thus, these results corroborated the finds from the literature, mainly after post-processing [30,38], in which the electrospinning and sterilization processes maintained the thermal properties of this polymer [15,30] and could have favored the annealing process. In theory, annealing occurs whenever the polymer is heated to a temperature above glass transition (T*_g_*) and below T_m_, in which annealing could eventually affect the mechanical properties [39]. For the PDO polymer, T*_g_* was measured before the sterilization process at 40.1 ± 2.2 °C and T_m_ at 102.36 ± 0.43 °C. Additionally, the parameters of the employed sterilization process via EtO gas were: 1. sterilizing agent (EtO 90%/CO_2_ 10%); 2. chamber temperature (50 ± 10 °C); 3. relative humidity (≥40%); and 4. total exposure time (2 h).

Overall, these analyses revealed that the thermal stability of the membranes did not change due to ethylene oxide sterilization, further indicating that it is an adequate process for PDO membrane sterilization. A validated sterilization process ensures the elimination of by-products generated during the process.

**Figure 2:**
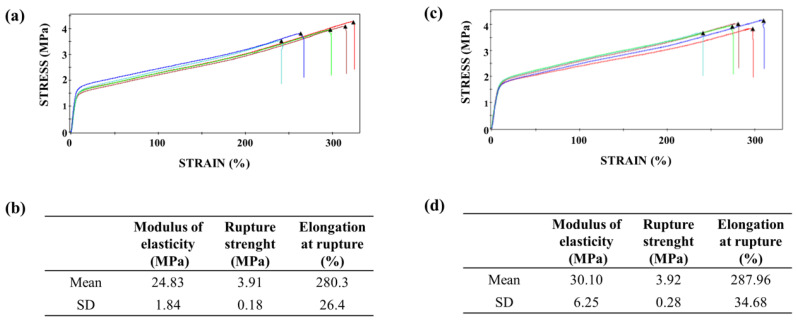
Mechanical traction properties. Tensile stress curves as a function of sample elongation, before (**a**) and after (**c**) the sterilization process. Mechanical properties in membrane traction, before (**b**) and after (**d**) the sterilization process. Test speed: 50 mm/min.

The mechanical properties, such as tensile strength (σ), elastic modulus (E), and strain at failure (ε) are summarized in Figure 2. The results obtained before and after the EtO sterilization process did not demonstrate significant statistical difference (Student’s *t*-test, *p* > 0.05). In addition, the PDO flexibility is improved as a result of the change of one ester linkage to an ether linkage with regards to other synthetic polymers; due to the unique ether bond, PDO has excellent flexibility [40]. Furthermore, the results suggest that sterilization via EtO favored the annealing process of the polymeric chain owing to the values measured to thermal (%Xc = 52.86 and T_m_ = 107.35 °C) and mechanical (E = 30.10 ± 6.25) properties. Therefore, the temperature used for the EtO process, 50 ± 10 °C per 2 h, might have benefited this annealing. The annealing process might determine the amorphous regions into ordered, i.e., the arrangement of molecular chains in the crystal [39], whereas the semicrystalline nature of PDO makes it possible to adjust the final and improved physical properties.

**Figure 3:**
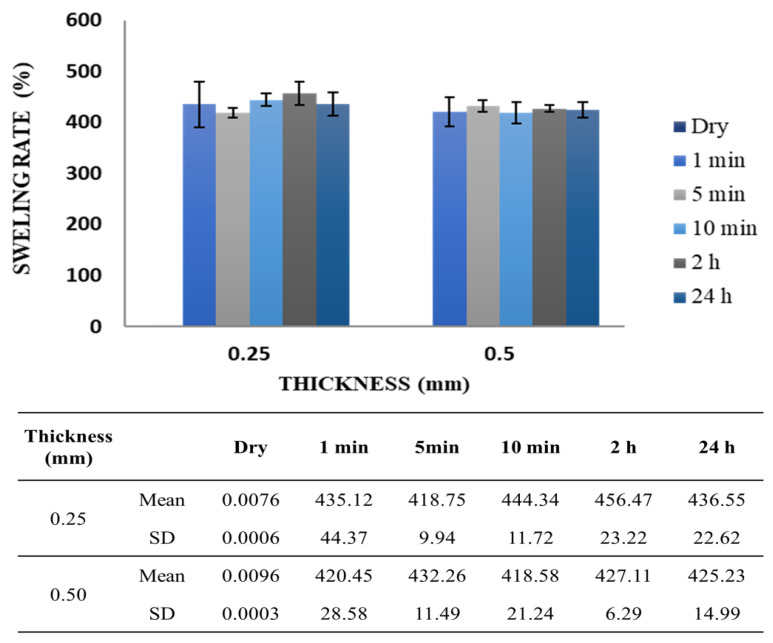
Swelling analysis of the 0.25- and 0.5- mm PDO membranes over time. The percentages were calculated in relation to the dry mass of the membrane.

Water absorption capacity for the PDO membranes was determined within 24 h. The mean percentage rate of swelling for the 0.25- and 0.5-mm membranes was 435.12 ± 44.37% and 420.45 ± 28.58% in relation to the dry mass of each type of membrane after only 1 min immersed (Figure 3). Throughout the analyzed time, swelling rate remained constant.

### 3.2. Integration and Migratory Capacity of hASCs on PDO-Based Membranes

**Figure 4:**
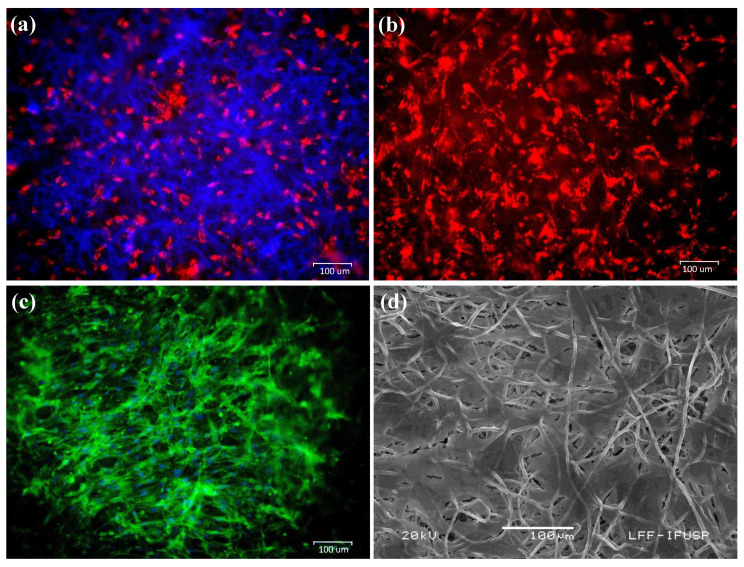
The hASCs seeded on PDO membranes. Cells were stained with (**a**) PHK26 and Hoechst solution 4 h after incubation on the membrane, (**b**) PHK26, and (**c**) FITC after 24 h of incubation. (**d**) SEM image of hASCs after 11 days on the membrane.

The hASCs were seeded on PDO-based membranes and analyzed via fluorescence microscopy after 4 and 24 h and via SEM on incubation day 11 (Figure 4). The images in Figure 4 illustrate that the hASCs were able to proliferate and integrate on the membranes.

After 24 h of incubation, the hASCs isolated from the culture supernatant were counted using an automated cell counter. Cell viability was between 74% and 94% on PDO membranes and 30% and 31% on collagen membranes for the replicated measurements. Meanwhile, in the control (0.9% NaCl), cell viability was 100% and 100% for the replicated measurements. Therefore, toxicity was calculated to be 26% and 6% (mean = 16%), and 70% and 69% (mean = 69.5%) for the PDO and collagen membranes, respectively. The statistical analysis indicated that the toxicity of the PDO membranes was 4.34 times lower than that of the collagen membranes (Student’s *t*-test, *p* < 0.05).

Considering the importance of stem-cell migration for the regeneration of injured tissues, the migratory capacity of the hASCs was evaluated after 21 days of cultivation on the tested biomaterials. After this period, it was verified that the hASCs were able to migrate from the PDO membranes at a higher rate than from the collagen membranes (Table 3).

### 3.3. Osteogenic Differentiation on PDO Membranes

**Figure 5:**
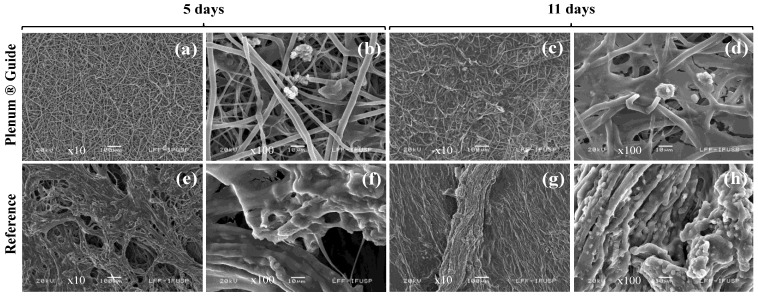
Osteogenic differentiation on the PDO membrane after incubation for (**a**,**b**) 5 and (**c**,**d**) 11 days. Osteogenic differentiation on the collagen membrane after incubation for (**e**,**f**) 5 and (**g**,**h**) 11 days.

The complete cell coating of the membrane fibers (with the osteogenic differentiated hASC) was observed in the collagen membranes, along with large morphological changes in the hASCs (Figure 5e–h). Meanwhile, on the PDO membranes, advanced osteogenic differentiation was observed, along with several calcification nodules (Figure 5a–d).

### 3.4. Toxicity and Clinical Evaluation

No signs of systemic toxicity or death were observed after the injection of PDO membrane extracts (Table S1—supplementary materials). Therefore, according to the acceptance score of the applied methodology, the material was considered safe.

The subchronic systemic toxicity assay was conducted with 23 animals in total. During the experimental period, three animals died; however, the deaths were not related to the test or to the reference (diarrhea and self-mutilation). Thus, the study was completed with 20 animals (four female tests and six female references; six male tests and four male references). There were no statistical differences in relation to the males’ body weights in the experimental and control groups (Table S2). A statistical difference in terms of weight gain was observed in the females: the control group exhibited a higher weight gain than the experimental group. Considering these results, as no relevant clinical signs were observed, and after the loss of two animals that may have interfered with the statistical results, this difference in weight gain was not considered a sign of toxicity related to the PDO membranes.

There was no statistical difference in the absolute and relative weights of the organs evaluated in the experimental and control groups (Table S3). Certain changes were observed in both experimental and control groups, such as lymphoid hyperplasia in the mesenteric lymph node, degeneration and necrosis in the popliteal lymph node, and hydropic degeneration in the liver (Table S4). Kidneys harvested from the animals in the experimental group exhibited mononuclear inflammation. However, none of the other organs (spleen, adrenal gland, and ovaries/testicles) presented any pathological alterations, both in the experimental and control groups. All the observed alterations are commonly associated with spontaneous pathologies in the analyzed species. Thus, no alterations related to the PDO membranes’ toxic effects were observed after implantation in the rabbit tibias after surgically inducing bone defects.

Local clinical aspects of the implantation sites were evaluated during necropsy (Table S5). One animal in the experimental group (01M) presented a bony callus at the tibia tuberosity at the implantation site. This may be due to the healing of a minor fracture. Moreover, a specimen presented non-repaired bone defects in the reference group (12F). Other clinical effects, such as edema, hematoma, encapsulation, and necrosis, were not observed.

### 3.5. Histological and Blood Evaluation

Bone neoformation in the experimental and control groups resulted in newly formed bone tissue, wherein bone neoformation was compounded with a lamellar bone with organized collagen fibers, osteocytes, Haversian canals, rare osteoblast rhymes, and a discrete amount of immature bone separated by cement lines. Minimal capillary proliferation was observed in one of the animals in the experimental group, which may be associated with discrete fibrosis and a minimal amount of fatty infiltrate at the implant site. Meanwhile, two of the animals in the experimental group presented a discrete area with fatty infiltrate. Minimal capillary proliferation was observed in only one of the animals in the control group, and it was associated with a discrete area of fatty infiltrate.

For the semiquantitative evaluation of the local bone reaction (Table 4), the mean of the experimental sites was 9.9, while the mean of the control sites was 8.6, which implies a tissue reaction index of 1.3 (not reactive compared with the control sample). PDO membranes induced a slightly higher amount of newly formed bone tissue and consequent reactions. In contrast, the control membranes had slightly smaller reactions and resulted in immature bone tissue (data per animal is presented in Table S6). However, this difference (1.3) is not relevant, and it is within the non-reactive classification (0.0 to 2.9) when compared to the control. Moreover, the PDO membranes did not result in any significant histological changes in the evaluated organs, indicating the absence of systemic toxicity. This implies that they can be considered non-reactive for bone tissues when compared to collagen membranes.

The blood count (erythrogram and leukogram), coagulogram, and liver and renal functions analysis indicated the absence of significant systemic changes in all animals (Tables S7–S11) [41].

## 4. Discussion

An ideal synthetic resorbable material must exhibit high porosity to promote tissue ingrowth and reduce the risk of tissue defect recurrence [42]. Therefore, the structural morphology and superficial topography of the PDO membrane examined in this study are similar to those of the ECM (Figure 1), in which the physicochemical and morphological properties facilitate the diffusion of biological fluids and cell adhesion [4]. Plenum^®^ Guide melting and crystallization temperatures are similar to that found in the literature for PDO polymer (Table 2) [17,38]. Moreover, this membrane presented elongation at rupture around 280%, which is a superior value to the one found in the literature data on commercial collagen membranes (15 up to 60%) [43,44,45] and indicates a more significant resistance necessary for its application (Figure 2). The membrane demonstrated a rapid water absorption capacity, reaching its maximum swelling limit in one minute of immersion, and kept the swelling rate constant throughout the studied period (Figure 3). This characteristic is of great value for the membrane’s application since after swelling the membrane and implantation in the patient there would be no dimensional change.

PDO is a safe synthetic graft material and is well known as a guide for tissue regeneration to stimulate bone and cartilage development in vivo [19,46,47,48,49]. PDS^®^ as an absorbable barrier material (polydioxanone, Johnson & Johnson, Ethicon GmbH & Co KG, Norderstedt, Germany) was used for periodontal regenerative surgery, which helped to eliminate a second surgical procedure for the removal of non-absorbable membranes [49]. Pretzl et al. [50] reported that, within the limitations of their study, the vertical gain and bony fill achieved using PDS barriers were stable in 68% of the evaluated intrabody defects ten years after GBR therapy. In contrast, there are several reports on complications resulting from the use of PDS. For example, infraorbital inflammation and persistent diplopia were observed eight weeks after operation [51]. In another study, a peri-implant reaction was observed in 60% of the test subjects after PDS resorption [52]. In an animal study, a peri-implant tissue reaction [53] and edemas [54] were observed around the degradation products.

Schmitt et al. [55] examined 0.25 mm-thick PDS^®^ films (Johnson & Johnson, Ethicon GmbH & Co KG, Norderstedt, Germany) as a cell-culture matrix for the second passage of in vitro cultured osteoblast-like cells. These films, which had low mechanical strength, only promoted cell adhesion and proliferation in certain areas. However, this study demonstrated that Plenum^®^ Guide membranes present superior rupture strength, modulus of elasticity, and elongation at rupture than PDS^®^ films [56,57], and allowed hASC ingrowth and integration on the biomaterial (Figure 4). Rowland et al. [4] suggested that the PDO scaffold fiber orientation influences the differentiation capacity of MSCs by manipulating their morphology [58]. Aligned scaffolds support greater adipogenic and osteogenic differentiation, while random scaffolds favor chondrogenesis. The structural morphology of Plenum^®^ Guide membranes, which is similar to that of the ECM, may explain the improved outcomes resulting from their use when compared with PDS [59,60].

Natural polymers attract special interest for tissue-engineering applications as they present biocompatible, biodegradable, and natural substrates on which cells can easily spread, attach, proliferate, and differentiate [61]. Bio-Gide^®^ (Geistlich Pharma AG, Wolhusen, Switzerland) is a natural double-sided porcine collagen (types I and III) membrane that has been used as a barrier for bone-tissue regeneration in bone-graft surgeries. Rupture elongation of this membrane is 46.8% [43], approximately six times less than that of on Plenum^®^ Guide membrane (Figure 2).

Collagen-based absorbable membranes result in high cell proliferation and cell viability concerning unrestricted somatic stem cells [62] and ASCs [63]. Our results show that hASCs can proliferate efficiently on Plenum^®^ Guide, where hASC migration was better on PDO membranes than on collagen membranes (Table 2). Furthermore, the toxicity of PDO membranes was at least four times lower than that of collagen membranes for this cell line. PDO-based biomaterials have been shown to facilitate the ingrowth and differentiation of hASCs in vitro [28,29]. Such materials better support adipocyte cell adhesion and viability than other polymer-based materials following implantation [64]. PDO membranes showed slightly better efficiency at hASC differentiation than collagen membranes, resulting in advanced osteogenic differentiation with several calcification points (Figure 5). These results indicate the potential application of the biomaterial as a scaffold for stem cells to engineer bone regeneration and to act as a barrier membrane.

There are several concerns regarding the use of natural polymers, including the lack of control over their physical and chemical characteristics and complex purification and sterilization processes. Moreover, they present a high risk of pathogenic/viral contamination and may lead to immune rejection when isolated from different sources [11,12]. Although collagen exhibits good potential for tissue regeneration and is often used in various biomedical applications, this biopolymer may induce a local inflammatory reaction after implantation [13]. In contrast, such a phenomenon is not observed with synthetic polymers due to their low immunogenicity [14,15]. In this study, no toxic effects and/or systemic changes were observed in animals treated with PDO membranes. Regardless of the difference found that was not relevant regarding the semiquantitative evaluation of the local bone reaction (Table 3), Plenum^®^ Guide resulted in a slightly higher amount of newly formed bone tissue in surgically induced bone defects. In contrast, the collagen membrane (Bio-Gide^®^) resulted in a small amount of immature bone tissue.

## 5. Conclusions

PDO-based membranes showed to be morphologically similar to ECM. Moreover, they demonstrated suitable mechanical properties and great dimensional stability after swelling and proved to be safe and efficient materials for use as resorbable membranes for bone regeneration. Their implantation in surgically induced bone defects resulted in a large amount of newly formed bone tissue. The excellent adhesion, growth, and migratory capacity of the hASCs on PDO-based membranes, along with their osteogenic differentiation, suggest that these membranes have significant potential for applications in tissue engineering and regenerative medicine.

## Figures and Tables

**Table 1:** Histological parameters and criteria to score and evaluate bone neoformation and reaction (per field, 400×, and a mean of five fields) and tissue response.

		Score				
		0	1	2	3	4
Bone neoformation/response	Osteoblast rhyme	0	Rare, 1–5	5–10	Infiltrate	Accumulated
	Osteoclasts	0	Rare, 1–5	5–10	Infiltrate	Accumulated
	Immature bone	0	Minimum	Middle	Moderate	Severe
	Non-mineralized osteoid matrix	0	Minimum	Middle	Moderate	Severe
	Necrosis	0	Minimum	Middle	Moderate	Severe
Tissue response	Neovascularization	0	Minimal capillary proliferation, focal 1–3 shoots	Groups of 4–7 capillaries with fibroblastic support structures	Broad bands of capillaries with support structures	Extensive range of capillaries with fibroblastic support structures
	Fibrosis	0	Narrow range	Moderate	Thick band	Extensive
	Fatty Infiltrate	0	Minimum amount of fat associated with fibrosis	Several layers of fat and accumulation of fibrous tissue	Elongated and wide fat cells on the implant site	Extensive fat that completely involves the implant

**Table 2:** DSC analysis before and after sterilization of the PDO membranes.

		T_m_ (°C)	∆H_m_ (J/g)	%Xc
Before sterilization	Mean	102.36	−74.62	56.39
	SD	0.43	4.19	0.70
After sterilization	Mean	107.35	−79.62	52.86
	SD	0.48	0.99	2.97

T_m_: melting temperature; ∆H_m_: melting enthalpy; %Xc: crystallinity degree.

**Table 3:** Rate of hASC migration from the PDO and collagen membranes. (−) No migration; (+) ~10^2^ cells/plaque; (++) ~10^4^ cells/plaque; (+++) ~10^5^ cells/plaque.

Membrane	Migration Rate
PDO	+++
Collagen	++

**Table 4:** Histological indices to evaluate tissue reaction.

Bone Neoformation/Response	Treated Group	Control Group
Osteoblast rhyme (sum of the group)	15	13
Osteoclasts (sum of the group)	1	0
Immature bone (sum of the group)	18	17
Non-mineralized osteoid matrix (sum of the group)	13	12
Necrosis (sum of the group)	0	0
Subtotal (2×)	94	84
Neovascularization (sum of the group)	2	1
Fibrosis (sum of the group)	1	0
Fatty infiltrate (sum of the group)	2	1
Subtotal	5	2
Group total	99	86
Group mean	9.9	8.6
Mean index ^1^	1.3	

^1^ Used to determine the tissue-reaction index.

## Data Availability

The data presented in this study are available on request from the corresponding author.

## Supplementary Materials

The following are available online at https://www.mdpi.com/article/10.3390/polym13111685/s1, Table S1: Clinical signs/observations to systemic toxicity assay; Table S2: Variation in the weight of the animals in post-operative conditions; Table S3 Absolute and relative weights of the liver, left kidney, and spleen in post-operative conditions; Table S4: Clinical evaluation of the control and treated groups after implantation (weekly); Table S5: Histological evaluation of tissue reaction after membrane implantation; Table S6: Evaluation of the implant location after necropsy; Table S7: Erythrograms of animals after implantation assay (mean ± SD); Table S8: Leukograms of animals after implantation assay (mean ± SD); Table S9: Blood count of animals after implantation assay (mean ± SD); Table S10: Blood biochemistry of animals after implantation assay: Renal function; Table S11: Blood biochemistry of animals after implantation assay: Liver function.

## References

[1] Retzepi M., Donos N. (2010). Guided bone regeneration: Biological principle and therapeutic applications. Clin. Oral Implants Res..

[2] Soldatos N.K., Stylianou P., Koidou P., Angelov N., Yukna R., Romanos G.E. (2017). Limitations and options using resorbable versus nonresorbable membranes for successful guided bone regeneration. Quintessence Int..

[3] Elgali I., Omar O., Dahlin C., Thomsen P. (2017). Guided bone regeneration: Materials and biological mechanisms revisited. Eur. J. Oral Sci..

[4] Rowland D.C.L., Aquilina T., Klein A., Hakimi O., Alexis-Mouthuy P., Carr A.J., Snelling S.J.B. (2016). A comparative evaluation of the effect of polymer chemistry and fiber orientation on mesenchymal stem cell differentiation. J. Biomed. Mater. Res. Part A.

[5] Boyan B.D., Hummert T.W., Dean D.D., Schwartz Z. (1996). Role of material surfaces in regulating bone and cartilage cell response. Biomaterials.

[6] Lu L., Zhu X., Valenzuela R.G., Currier B.L., Yaszemski M.J. (2001). Biodegradable polymer scaffolds for cartilage tissue engineering. Clin. Orthop. Relat. Res..

[7] Li X., Wang M., Jing X., Guo W., Hao C., Zhang Y., Gao S., Chen M., Zhang Z., Zhang X. (2018). Bone marrow and adipose tissue-derived mesenchymal stem cells: Characterization, differentiation, and applications in cartilage tissue engineering. Crit. Rev. Euk. Gene Exp..

[8] Freed L.E., Grande D.A., Lingbin Z., Emmanual J., Marquis J.C., Langer R. (1994). Joint resurfacing using allograft chondrocytes and synthetic biodegradable polymer scaffolds. J. Biomed. Mater. Res..

[9] Lee C.H., Single A., Lee Y. (2001). Biomedical applications of collagen. Int. J. Pharm..

[10] Badylak S.F. (2007). The extracellular matrix as a biologic scaffold material. Biomaterials.

[11] Dawson E., Mapili G., Erickson K., Taqvi S., Roy K. (2008). Biomaterials for stem cell differentiation. Adv. Drug Deliv. Rev..

[12] Liu X., Ma P.X. (2004). Polymeric scaffolds for bone tissue engineering. Ann. Biomed. Eng..

[13] Lucke S., Hoene A., Walschus U., Kob A., Pissarek J.W., Schlosser M. (2015). Acute and chronic local inflammatory reaction after implantation of different extracellular porcine dermis collagen matrices in rats. Biomed Res. Int..

[14] Kock L., van Donkelaar C.C., Ito K. (2012). Tissue engineering of functional articular cartilage: The current status. Cell Tissue Res..

[15] Boland E.D., Coleman B.D., Barnes C.P., Simpson D.G., Wnek G.E., Bowlin G.L. (2005). Electrospinning polydioxanone for biomedical applications. Acta Biomater..

[16] Garg K., Pullen N.A., Oskeritzian C.A., Ryan J.J., Bowlin G.L. (2013). Macrophage functional polarization (M1/M2) in response to varying fiber and pore dimensions of electrospun scaffolds. Biomaterials.

[17] Goonoo N., Jeetah R., Bhaw-Luximon A., Jhurry D. (2015). Polydioxanone-based bio-materials for tissue engineering and drug/gene delivery applications. Eur. J. Pharm. Biopharm..

[18] Song S.J., Shin Y.C., Kim S.E., Kwon I.K., Lee J.H., Hyon S.H., Han D.W., Kim B. (2018). Aligned laminin core-polydioxanone/collagen shell fiber matrices effective for neuritogenesis. Sci. Rep..

[19] Boenisch M., Tamás H., Nolst Trenité G.J. (2003). Influence of polydioxanone foil on growing septal cartilage after surgery in an animal model: New aspects of cartilage healing and regeneration (preliminary results). Arch. Facial Plast. Surg..

[20] Rodriguez I.A., Madurantakam P.A., McCool J.M., Sell S.A., Yang H., Moon P.C., Bowlin G.L. (2012). Mineralization potential of electrospun PDO-hydroxyapatite-fibrinogen blended scaffolds. Int. J. Biomater..

[21] Kim T.H., Oh S.H., Chun S.Y., Lee J.H. (2014). Bone morphogenetic proteins-immobilized polydioxanone porous particles as an artificial bone graft. J. Biomed. Mater. Res. Part A.

[22] Caplan A.I. (1990). Mesenchymal stem cells. J. Orthop. Res..

[23] Pittenger M.F., Mackay A.M., Beck S.C., Jaiswal R.K., Mosca J.D., Moorman M.A., Simonetti D.W., Craig S., Marshak D.R., Pittenger M.F. (2016). Multilineage potential of adult human mesenchymal stem cells. Science.

[24] Levi B., Longaker M.T. (2011). Concise review: Adipose-derived stromal cells for skeletal regenerative medicine. Stem Cells.

[25] Travničková M., Bačáková L. (2018). Application of adult mesenchymal stem cells in bone and vascular tissue engineering. Physiol. Res..

[26] Burdick J.A., Vunjak-Novakovic G. (2009). Engineered microenvironments for controlled stem cell differentiation. Tissue Eng. Part A.

[27] Meng X., Leslie P., Zhang Y., Dong J. (2014). Stem cells in a three-dimensional scaffold environment. Springerplus.

[28] Bottino M.C., Kamocki K., Yassen G.H., Platt J.A., Vail M.M., Ehrlich Y., Spolnik K.J., Gregory R.L. (2013). Bioactive nanofibrous scaffolds for regenerative endodontics. J. Dent. Res..

[29] Pontailler M., Illangakoon E., Williams G.R., Marijon C., Bellamy V., Balvay D., Autret G., Vanneaux V., Larghero J., Planat-Benard V. (2015). Polymer-based reconstruction of the inferior vena cava in rat: Stem cells or RGD peptide?. Tissue Eng. Part A.

[30] Goonoo N., Bhaw-Luximon A., Rodriguez I.A., Wesner D., Schönherr H., Bowlin G.L., Jhurry D. (2014). Poly(ester-ether)s: II. Properties of electrospun nanofibres from polydioxanone and poly(methyl dioxanone) blends and human fibroblast cellular proliferation. Biomater. Sci..

[31] (2018). ASTM D882-18, Standard Test Method for Tensile Properties of Thin Plastic Sheeting.

[32] (2018). ASTM D570-98, Standard Test Method for Water Absorption of Plastics.

[33] Lizier N.F., Kerkis A., Gomes C.M., Hebling J., Oliveira C.F., Caplan A.I., Kerkis I. (2012). Scaling-Up of Dental Pulp Stem Cells Isolated from Multiple Niches. PLoS ONE.

[34] Debnath T., Chelluri L.K. (2018). Standardization and quality assessment for clinical grade mesenchymal stem cells from human adipose tissue. Hematol. Transfus. Cell Ther..

[35] Sundelacruz S., Kaplan D.L. (2009). Stem cell- and scaffold-based tissue engineering approaches to osteochondral regenerative medicine. Semin. Cell Dev. Biol..

[36] (2017). ISO 10993-11: Biological Evaluation of Medical Devices. Part 11: Tests for Systemic Toxicity.

[37] (2010). ISO 10993-6: Biological Evaluation of Medical Devices. Part 6: Tests for Local Effects after Implantation.

[38] Sabino M.A., González S., Márquez L., Feijoo J.L. (2000). Study of the hydrolytic degradation of polydioxanone PPDX. Polym. Degrad. Stab..

[39] Liu X., Feng S., Wang X., Qi J., Lei D., Li Y., Bai W. (2020). Tuning the mechanical properties and degradation properties of polydioxanone isothermal annealing. Turk. J. Chem..

[40] Hutmacher D., Hürzeler M.B., Schliephake H. (1996). A review of material properties of biodegradable and bioresorbable polymers and devices for GTR and GBR applications. Int. J. Oral Maxillofac. Implants.

[41] Suckow M.A., Stevens K.A., Wilson R.P. (2012). The Laboratory Rabbit, Guinea Pig, Hamster, and Other Rodents.

[42] Fatkhudinov T., Tsedik L., Arutyunyan I., Lokhonina A., Makarov A., Korshunov A., Elchaninov A., Kananykhina E., Vasyukova O., Usman N. (2019). Evaluation of resorbable polydioxanone and polyglycolic acid meshes in a rat model of ventral hernia repair. J. Biomed. Mater. Res. Part B Appl. Biomater..

[43] Ortolani E., Quadrini F., Bellisario D., Santo L., Polimeni A., Santarsiero A. (2015). Mechanical qualification of collagen membranes used in dentistry. Ann. Ist. Super Sanità.

[44] Coïc M., Placet V., Jacquet E., Meyer C. (2010). Propriétés mécaniques des membranes de collagne. Rev. Stomatol. Chir. Maxillofac..

[45] Charulatha V., Rajaram A. (2003). Influence of different crosslinking treatments on the physical properties of collagen membranes. Biomaterials.

[46] Becker S.T., Terheyden H., Fabel M., Kandzia C., Möller B., Wiltfang J. (2010). Comparison of collagen membranes and polydioxanone for reconstruction of the orbital floor after fractures. J. Craniofac. Surg..

[47] Rimmer J., Ferguson L.M., Saleh H.A. (2012). Versatile applications of the polydioxanone plate in rhinoplasty and septal surgery. Arch. Facial Plast. Surg..

[48] San-Marina S., Sharma A., Voss S.G., Janus J.R., Hamilton G.S. (2017). Assessment of scaffolding properties for chondrogenic differentiation of adipose-derived mesenchymal stem cells in nasal reconstruction. JAMA Facial Plast. Surg..

[49] Dörfer C.E., Kim T.S., Steinbrenner H., Holle R., Eickholz P. (2000). Regenerative periodontal surgery in interproximal intrabony defects with biodegradable barriers. J. Clin. Periodontol..

[50] Pretzl B., Kim T.S., Steinbrenner H., Dörfer C., Himmer K., Eickholz P. (2009). Guided tissue regeneration with bioabsorbable barriers III 10-year results in infrabony defects. J. Clin. Periodontol..

[51] Baumann A., Burggasser G., Gauss N., Ewers R. (2002). Orbital floor reconstruction with an alloplastic resorbable polydioxanone sheet. Int. J. Oral Maxillofac. Surg..

[52] Kontio R., Suuronen R., Salonen O., Paukku P., Konttinen Y.T., Lindqvist C. (2001). Effectiveness of operative treatment of internal orbital wall fracture with polydioxanone implant. Int. J. Oral Maxillofac. Surg..

[53] Merten H.A., Luhr H.G. (1994). Resorbable synthetics (PDS foils) for bridging extensive orbital wall defects in an animal experiment comparison. Fortschr Kiefer Gesichtschir.

[54] Kontio R., Ruuttila P., Lindroos L., Suuronen R., Salo A., Lindqvist C., Virtanen I., Konttinen Y.T. (2005). Biodegradable polydioxanone and poly (L/D) lactide implants: An experimental study on peri-implant tissue response. Int. J. Oral Maxillofac. Surg..

[55] Schmitt S.C., Wiedmann-Al-Ahmad M., Kuschnierz J., Al-Ahmad A., Huebner U., Schmelzeisen R., Gutwald R. (2008). Comparative in vitro study of the proliferation and growth of ovine osteoblast-like cells on various alloplastic biomaterials manufactured for augmentation and reconstruction of tissue or bone defects. J. Mater. Sci. Mater. Med..

[56] Birkenfeld F., Behrens E., Kern M., Gassling V., Wiltfang J. (2015). Mechanical properties of collagen membranes: Are they sufficient for orbital floor reconstructions?. J. Cranio Maxillofac. Surg..

[57] Birkenfeld F., Flörke C., Behrens E., Rohnen M., Kern M., Gassling V., Wiltfang J. (2015). Mechanical properties of collagen membranes modified with pores—are they still sufficient for orbital floor reconstruction?. Br. J. Oral Maxillofac. Surg..

[58] McBeath R., Pirone D.M., Nelson C.M., Bhadriraju K., Chen C.S. (2004). Cell shape, cytoskeletal tension, and RhoA regulate stem cell lineage commitment. Dev. Cell.

[59] Bhowmick S., Scharnweber D., Koul V. (2016). Co-cultivation of keratinocyte-human mesenchymal stem cell (hMSC) on sericin loaded electrospun nanofibrous composite scaffold (cationic gelatin/hyaluronan/chondroitin sulfate) stimulates epithelial differentiation in hMSCs: In vitro study. Biomaterials.

[60] Kendal A., Snelling S., Dakin S., Stace E., Mouthuy P.A., Carr A. (2017). Resorbable electrospun polydioxanone fibres modify the behaviour of cells from both healthy and diseased human tendons. Eur. Cells Mater..

[61] Fleischer S., Miller J., Hurowitz H., Shapira A., Dvir T. (2015). Effect of fiber diameter on the assembly of functional 3D cardiac patches. Nanotechnology.

[62] Schorn L., Handschel J., Lommen J., Von Beck F.P., Depprich R., Kübler N., Holtmann H. (2019). Evaluation of biocompatibility of different membrane surfaces using unrestricted somatic stem cells. In Vivo.

[63] Açil Y., Zhang X., Nitsche T., Möller B., Gassling V., Wiltfang J., Gierloff M. (2014). Effects of different scaffolds on rat adipose tissue derived stroma cells. J. Cranio Maxillofac. Surg..

[64] Trojahn Kølle S.F., Oliveri R.S., Glovinski P.V., Elberg J.J., Fischer-Nielsen A., Drzewiecki K.T. (2012). Importance of mesenchymal stem cells in autologous fat grafting: A systematic review of existing studies. J. Plast. Surg. Hand Surg..

