# Supplementary material for: Polydioxanone-Based Membranes for Bone Regeneration
Source: Polymers (Basel). 2021 May 21;13(11):1685. doi: 10.3390/polym13111685 (PMC8196877; doi:10.3390/polym13111685)
Supplement: Supplementary file 1 [file polymers-13-01685-s001.zip › polymers-1203010-supplementary.pdf]

polymers

MDPI

Supplementary Materials: Polydioxanone-Based Membranes for Bone Regeneration

Sybele Saska, Livia Pilatti, Edvaldo Santos de Sousa Silva, Magda Aline Nagasawa, Diana Câmara, Nelson Lizier, Eduardo Finger, Marta Dyszkiewicz Konwinska, Bartosz Kempisty, Samy Tunchel, Alberto Blay and Jamil Awad Shibli

Table S1. Clinical signs/observations to systemic toxicity assay.

Group	Animal	Clinical evaluation 24 hours	48 hours	72 hours	Mortality
Treated	1	NO	NO	NO	0/5 (0%)
	2	NO	NO	NO	
	3	NO	NO	NO	
	4	NO	NO	NO	
	5	NO	NO	NO	
Control	1	NO	NO	NO	0/5 (0%)
	2	NO	NO	NO	
	3	NO	NO	NO	
	4	NO	NO	NO	
	5	NO	NO	NO	

NO: no observation.

Table S2. Variation in the weight of the animals in post-operative conditions.

Sex	Group	Initial weight (g)	Final weight (g)	Variation[1]
Female	PDO	2,482.3 ± 293.6 (N = 6)	3,898.5 ± 329.9 (N = 4)	1,295.5 ± 295.7 (N = 4)
	Collagen	2,457.3 ± 325.6 (N = 6)	4,250.7 ± 329.1 (N = 6)	1,793.3 ± 358.8 (N = 6)
	P value	0.63[2]	0.14	0.05
Male	PDO	2,579.7 ± 106.3 (N = 6)	3,698.3 ± 228.8 (N = 6)	1,118.7 ± 298.4 (N = 6)
	Collagen	2,685.6 ± 210.0 (N = 5)	3,983.0 ± 474.8 (N = 4)	1,244.0 ± 360.8 (N = 4)
	P value	0.30	0.23	0.57

N: Number of animals in the group (N of the animals to final weight was different to the Female/PDO and Male/Collagen groups because during the experimental period, three animals died).

[1]Variation = final weight – initial weight.

[2]Mann-Whitney test.

Table S3. Absolute and relative weights of the liver, left kidney, and spleen in post-operative conditions.

Organ		Female PDO membrane (N = 4)	Female Collagen membrane (N = 6)	*P* value	Male PDO membrane (N = 6)	Male Collagen membrane (N = 4)	*P* value
Liver	AW (g)	99.40 ± 19.24	106.71 ± 35.97	0.72	97.73 ± 19.67	119.75 ± 20.49	0.13
	RW (g)	2.540 ± 0.381	2.485 ± 0.75	0.90	2.641 ± 0.504	2.993 ± 0.165	0.22
	AW (g)	1.310 ± 0.840	1.000 ± 0.21	0.51	0.89 ± 0.43	0.87 ± 0.22	0.95

Left Kidney	RW (g)	0.035 ± 0.025	0.023 ± 0.005	0.43	0.024 ± 0.012	0.023 ± 0.08	0.82
Spleen	AW (g)	8.37 ± 0.75	8.49 ± 1.23	0.87	8.25 ± 1.22	9.08 ± 0.86	0.28
	RW (g)	0.215 ± 0.021	0.199 ± 0.019	0.24	0.222 ± 0.260	0.230 ± 0.032	0.70

N: number of animals in the group; AW: Absolute weight; RW: Relative weight (organ weight/final body weight × 100).

Table S4. Clinical evaluation of the control and treated groups after implantation (weekly).

Group	Animal	Day													
		0	7	14	21	28	35	42	49	56	63	70	77	84	90
Treated	01F[1]	NO	-	-	-	-	-	-	-	-	-	-	-	-	-
	02F	NO	NO	NO	NO	NO	NO	NO	NO	NO	NO	NO	NO	NO	NO
	03F	NO	NO	NO	NO	NO	NO	NO	NO	NO	NO	NO	NO	NO	NO
	04F	NO	11	NO	NO	NO	NO	NO	NO	NO	NO	NO	NO	NO	NO
	05F	NO	14	NO	NO	NO	NO	NO	NO	NO	NO	NO	NO	NO	NO
	06F[2]	NO	14	-	-	-	-	-	-	-	-	-	-	-	-
	01M	NO	NO	NO	NO	NO	NO	3[3]	NO	NO	NO	NO	NO	NO	NO
	02M	NO	NO	NO	NO	NO	NO	NO	NO	NO	NO	NO	NO	NO	NO
	03M	NO	NO	7	NO	NO	NO	NO	NO	NO	NO	NO	NO	NO	NO
	04M	NO	NO	NO	NO	NO	NO	3[3]	NO	NO	NO	NO	NO	NO	NO
	05M	NO	NO	NO	NO	NO	NO	NO	NO	NO	NO	NO	NO	NO	NO
	06M	NO	NO	NO	NO	NO	NO	NO	NO	NO	NO	NO	NO	NO	NO
Control	07F	NO	7	7	NO	NO	NO	NO	NO	NO	NO	NO	NO	NO	NO
	08F	NO	NO	NO	NO	NO	NO	NO	NO	NO	NO	NO	NO	NO	NO
	09F	NO	NO	NO	NO	NO	NO	NO	NO	NO	NO	NO	NO	NO	NO
	10F	NO	NO	NO	NO	NO	NO	NO	NO	NO	NO	NO	NO	NO	NO
	11F	NO	NO	NO	NO	NO	NO	NO	NO	NO	NO	NO	NO	NO	NO
	12F	NO	14	NO	NO	NO	NO	NO	NO	NO	NO	NO	NO	NO	NO
	07M	NO	NO	NO	NO	NO	NO	NO	NO	NO	NO	NO	NO	NO	NO
	08M	NO	NO	NO	NO	NO	NO	NO	NO	NO	NO	NO	NO	NO	NO
	09M	NO	7	7	NO	NO	NO	NO	NO	NO	NO	15	15	15	15
	10M	NO	NO	NO	NO	NO	NO	NO	NO	NO	NO	NO	NO	NO	NO
	11M	NO	NO	NO	NO	NO	NO	NO	3[4]	-	-	-	-	-	-

Clinical signs: NO – nothing observed; 1 - Death; 2 – Seizure; 3 – Mutilation; 4 – Prostration; 5 – Ataxia; 6 – Tremors; 7 – Local inflammation; 8 – Dyspnea; 9 – Tearing; 10 – Salivation; 11 – Diarrhea; 12 – Piloerection; 13 – Cachexia; 14 – Hematoma at implant site; 15 – Ocular secretion. / [1] Animal presented diarrhea and died on day 5 of the experimental period; [2] Animal presented diarrhea and died on the 11th of the experimental period; [3] Mutilation in the contralateral limb to the implant, [4] Animal presented an infected wound due to limb self-mutilation and was euthanized for humanitarian reasons on the 49th of the experimental period.

Table S5. Histological evaluation of tissue reaction after membrane implantation.

	Animals																			
	Treated										Control									
	02F	03F	04F	05F	01M	02M	03M	04M	05M	06M	07F	08F	09F	10F	11F	12F	07M	08M	09M	10M
Osteoblast rhyme	1	1	2	2	2	1	1	2	2	1	1	1	1	1	1	2	1	1	2	2
Osteoclasts	0	0	0	1	0	0	0	0	0	0	0	0	0	0	0	0	0	0	0	0
Immature Bone	2	2	2	2	2	1	2	2	2	1	2	2	2	2	1	3	1	1	1	2
Non-mineralized osteoid matrix	1	1	1	3	1	1	1	2	1	1	1	2	1	1	1	2	1	1	1	1
Necrosis	0	0	0	0	0	0	0	0	0	0	0	0	0	0	0	0	0	0	0	0
Subtotal (2x)	8	8	10	16	10	6	8	12	10	6	8	10	8	8	6	14	6	6	8	10
Neovascularization	1	0	0	1	0	0	0	0	0	0	0	0	0	0	0	1	0	0	0	0
Fibrosis	0	0	0	1	0	0	0	0	0	0	0	0	0	0	0	0	0	0	0	0

Fatty infiltrate	0	0	1	1	0	0	0	0	0	0	0	0	0	0	0	1	0	0	0	0
Subtotal	1	0	1	3	0	0	0	0	0	0	0	0	0	0	0	2	0	0	0	0
Total	9	8	11	19	10	6	8	12	10	6	8	10	8	8	6	16	6	6	8	10
Group Total					99											86				
Group Mean					9.9											8.6				
Mean Index[1]											1.3									

[1] Used to determine the tissue-reaction index.

Table S6. Evaluation of the implant location after necropsy.

Group	Animal	Macroscopic alterations	Group	Animal	Macroscopic alterations
PDO membrane	02F	NO	Collagen membrane	07F	NO
	03F	NO		08F	NO
	04F	NO		09F	NO
	05F	NO		10F	NO
	01M	E		11F	NO
	02M	NO		12F	NO[1]
	03M	NO		07M	NO
	04M	NO		08M	NO
	05M	NO		09M	NO
	06M	NO		10M	NO

NO: nothing observed; E: bony callus in tibia tuberosity.

[1]Non-repaired bone defect.

Table S7. Erythrograms of animals after implantation assay (mean ± SD).

Group	Parameters and reference values						
	Erythrocytes (5.1 to 7.9 million/mm³)	Hemoglobin (10 to 17.4 g/dL)	Hematocyte (33 to 50%)	ACV (57.8 to 66.5 u3)	MCH (17.1 to 23.5 pg)	CMCH (29 to 37 g/dL)	Total Protein (5.4 to 8.5 g/dL)
Treated	6.04±1.09	12.61±1.92	37.94±6.19	63.12±2.93	21.04±1.29	33.30±0.61	5.80±0.65
Control	6.16±0.37	13.09±1.41	40.04±2.78	65.08±3.37	21.30±2.31	32.70±2.92	5.86±0.21
P value	0.36[1]	0.53	0.55[1]	0.13[1]	0.76	0.54	0.79

SD: Standard Deviation; ACV: Average Corpuscular Volume; MCH: Middle Corpuscular Hemoglobin; CMCH: Concentration of Middle Corpuscular Hemoglobin.

[1] Mann-Whitney Test.

Table S8. Leukograms of animals after implantation assay (mean ± SD).

Group	Parameters and reference values							
	Leukocytes (5.2 to 12.5 ml/mm³)	Metamyelocytes (0%)	Neutrophils (20 - 75%)	Eosinophils (1 to 4%)	Basophils (1 to 7%)	Lymphocytes (30 to 85%)	Monocytes (1 to 4%)	Platelets (50 to 650 ml/mm³)
Treated	5.11±1.67	0.00±0.00	42.40±16.53	0.20±0.63	0.00±0.00	56.20±16.13	1.20±0.79	223.30±84,39
Control	6.38±1.16	0.00±0.00	45.00±13.15	0.40±0.70	0.00±0.00	53.10±12.87	1.50±0.71	267.90±54.81
P value	0.06	-	0.70	0.50[1]	-	0.64	0.55[1]	0.18

SD: Standard Deviation.

[1] Mann-Whitney Test.

Table S9. Blood count of animals after implantation assay (mean ± SD).

Group	PT	APTT
Treated	6.56±0.13	21.03±2.74
Control	7.09±0.64	23.47±6.71

P value	0.02[1]	0.32

SD: Standard Deviation; PT: prothrombin time; APTT: activated partial thromboplastin time.

[1] Mann-Whitney Test.

Table S10. Blood biochemistry of animals after implantation assay: Renal Function.

	**Parameters and reference values**					
	**Urea (13 to 52 mg/dL)**	**Creatinine (0.80 to 1.80 mg/dL)**	**Sodium (138 to 148 mmol/L)**	**Potassium (3.3 to 6.9 mmol/L)**	**Chlorine (92 to 112 mmol/L)**	**Calcium (5.6 to 12.0 mg/dL)**
**Treated**	48.05±4.74	1.20±0.23	146.32±2.43	4.97±0.47	95.06±3.30	13.51±0.36
Control	47.09±7.32	1.13±0.17	147.15±1.83	4.76±0.82	96.41±4.33	13.49±0.35
P value	0.73	0.46	0.40	0.50	0.44	0.90

SD: Standard Deviation.

Table S11. Blood biochemistry of animals after implantation assay: Liver function.

	**Parameters and reference values**							
	**ALT (31 to 60 U/L)**	**Phosphatase (90 to 145 U/L)**	**AST (42 to 98 U/L)**	**GGT (4 to 12 U/L)**	**Bilirubin (0.3 to 0.8 mg/dL)**	**Total protein (5.4 to 6.8 g/dL)**	**Albumin (3.4 to 4.6 g/dL)**	**Globulin (1.5 to 2.9 g/dL)**
Treated	64.90±12.17	59.80±31.08	38.20±13.36	5.70±1.06	0.12±0.06	5.24±0.28	3.32±0.20	1.88±0.23
Control	67.70±16.26	62.40±23.82	36.60±8.14	5.40±1.17	0.11±0.03	5.16±0.21	3.32±0.14	1.84±0.13
P value	0.67	0.84	0.94[1]	0.57[1]	0.97[1]	0.50	1.00	0.65

SD: Standard Deviation; ALT: alanine aminotransferase; AST: aspartate aminotransferase; GGT: Gamma Glutamyl Transferase.

[1] Mann-Whitney Test.
